# Single-nucleus transcriptomic atlas of sexually dimorphic molecular responses to sub-chronic variable stress in the mouse hippocampus

**DOI:** 10.1038/s41398-026-04202-3

**Published:** 2026-06-24

**Authors:** Liang Liang, Yu-pei Yuan, Chun-lei Chang, Jing Zhang, Chen Liang

**Affiliations:** 1 Department of Psychology, The Fourth Affiliated Hospital of Xinjiang Medical University, Urumqi, Xinjiang China; 2 Department of Cardiology, Renmin Hospital of Wuhan University, Hubei Zhang Road (formerly Ziyang Road), Wuchang District No. 99, Jiefang Road 238, Wuhan, Hubei China; 3 The Fourth Clinical Medical College of Xinjiang Medical University, Urumqi, Xinjiang China

**Keywords:** Depression, Genomics

## Abstract

**Background:**

Major depressive disorder (MDD) exhibits a higher prevalence in women, yet the underlying cellular mechanisms for this clinical disparity remain elusive. To investigate sex-specific molecular mechanisms, we profiled sex-specific molecular responses to sub-chronic stress in the mouse hippocampus at single-nucleus resolution.

**Methods:**

Male and female C57BL/6N mice were exposed to sub-chronic variable stress (SCVS). Anhedonia was assessed via sucrose preference and novelty-suppressed feeding. We employed single-nucleus RNA sequencing (snRNA-seq) to map sex-specific hippocampal responses to sub-chronic variable stress (SCVS) in mice.

**Results:**

Female mice displayed significantly exacerbated anhedonia and selective depletion of plasma serotonin (5-HT) following SCVS exposure, contrasting with minimal behavioral changes in males. Single-nucleus RNA sequencing of 31,256 hippocampal cells revealed profound sex-dimorphic transcriptional reprogramming: females exhibited a 3-fold greater number of differentially expressed genes (DEGs) than males with minimal overlap. Key mechanistic findings included: (1) Females exhibited oligodendrocyte-specific upregulation of mitochondrial genes (mt-Atp6, mt-Co3), increasing oxidative stress susceptibility. Concurrently, stress depleted their baseline oligodendrocyte predominance, potentially disrupting hippocampal synchrony underlying anhedonia.; (2) Females exhibit broad Mef2c upregulation, while males show astrocyte/cholinergic neuron NRG-1 upregulation and astrocyte Slc6a11 downregulation; (3) Females display suppressed oxytocin signaling and coordinated dampening of glutamatergic signaling, astrocytic K⁺ buffering, and cell adhesion; males exhibit dysregulated kinase/phosphatase activity.

**Conclusion:**

This study presents the single-nucleus atlas of hippocampal sexual dimorphism in stress response, identifying oligodendrocyte mitochondrial dysfunction, pan-cellular Mef2c upregulation, and oxytocin suppression as potential female-specific therapeutic targets for depression. Our findings reveal fundamental sex-divergent molecular adaptations to stress, providing a roadmap for novel, sex-stratified treatments for MDD.

## Introduction

Major depressive disorder (MDD) is a pressing global public health concern that exhibits a well-documented and consistent sexual dimorphism, with epidemiological studies reporting a approximately twofold higher incidence in women compared to men [1, 2]. This persistent disparity across diverse populations strongly suggests the contribution of intrinsic biological factors. Consequently, there is a critical need to move beyond phenomenological observations and delineate the fundamental sex-specific molecular mechanisms that underlie stress susceptibility, a key etiological driver of MDD. Chronic stress, a well-recognized etiological driver of MDD, elicits markedly distinct pathophysiological responses in preclinical models along sexual lines [3]. Female rodents, for instance, display heightened susceptibility to the development of anhedonia—a core depressive phenotype characterized by a profound diminishment in reward processing [4, 5]—and exhibit a distinct dysregulation of the hypothalamic-pituitary-adrenal (HPA) axis compared to males, a key neuroendocrine system involved in stress regulation [6–8].

The hippocampus, a brain region central to the integration of stress signals, the regulation of emotional responses, and the mediation of cognitive functions, undergoes sexually dimorphic structural alterations in the context of depression [9]. These include female-specific reductions in neurogenesis and male-predominant patterns of dendritic remodeling, which collectively contribute to the observed behavioral differences [10, 11]. However, despite these compelling observations, the cell type-resolved molecular mechanisms that underlie the divergent hippocampal adaptations to stress between the sexes remain poorly understood. This knowledge gap is particularly pronounced with respect to the contributions of non-neuronal cells, such as glia, and subcellular compartments, including mitochondria, which have increasingly been recognized as crucial players in neural function and dysfunction [12]. While this study focuses on the sexually dimorphic hippocampus in stress response, we acknowledge that depressive circuits extend to other regions. In particular, deficient glutamate transmission in the prefrontal cortex is strongly implicated in anhedonia pathogenesis.

Existing mechanistic insights into MDD have largely been derived from two types of studies: bulk-tissue analyses, which average molecular signals across diverse cell populations, thereby masking cell type-specific changes [13, 14], and male-focused single-nucleus investigations, which fail to capture the critical interactions between sex and cell type [15, 16]. Bulk transcriptomic studies have identified sex-biased gene expression patterns in the stressed hippocampus, such as female-specific downregulation of the brain-derived neurotrophic factor (Bdnf) gene, but are unable to resolve the specialized responses of distinct cellular subpopulations [17]. While emerging single-nucleus RNA sequencing (snRNA-seq) approaches offer the potential for high-resolution dissection of neural heterogeneity [18, 19], and a growing number of pioneering studies have begun to incorporate comparisons between sexes under stress conditions or in MDD [20–22], several critical knowledge gaps remain. Specifically, the cell type-resolved mechanisms underlying female vulnerability in the hippocampus, particularly involving non-neuronal cells and mitochondrial genomics in the context of sub-chronic variable stress, have not been systematically elucidated.

As a result, three fundamental knowledge gaps persist in the field. First, the role of glial subtypes—including astrocytes, oligodendrocytes, and microglia—in orchestrating sex-dimorphic stress responses remains largely uncharacterized [23]. These cells are key regulators of neuroinflammation, myelination, and metabolic support within the central nervous system, and their contributions to sexual dimorphism in depression susceptibility are likely substantial [24–26]. Second, the potential involvement of mitochondrial genomics, particularly maternally inherited mitochondrial DNA (mtDNA)-encoded genes, in mediating female-specific susceptibility to oxidative stress in the context of MDD has not been adequately explored [27, 28]. Mitochondria are critical for energy production and are major sources of reactive oxygen species, making them plausible candidates for involvement in stress-related pathology [29, 30]. Third, the extent to which neuronal circuit-specific plasticity—such as differences in glutamatergic versus GABAergic neurotransmission—drives the divergent behavioral outcomes observed between males and females following stress exposure remains unclear [31].

To address these critical knowledge gaps, we sought to systematically characterize the sex-specific molecular landscape of the stress response. We employed the subchronic variable stress (SCVS) paradigm, which has been validated to induce maladaptive responses in rodents through the alternating application of physiological and psychological stressors over a six-day period. This paradigm exerts a more rapid impact on female rodents compared to males, potentially representing a maladaptive mechanism [32]. This model offers distinct advantages over chronic unpredictable stress models [33], as it rapidly elicits anhedonia while minimizing stressor habituation, thereby enabling precise temporal alignment of molecular and behavioral analyses. We coupled this stress paradigm with high-throughput snRNA-seq using the 10x Genomics Chromium platform, which allows for the comprehensive profiling of gene expression at the single-nucleus level.

The primary goal of this study was not merely to confirm established behavioral differences, but to determine whether males and females mount fundamentally distinct molecular adaptations to stress within the hippocampus, even in the presence of overlapping behavioral phenotypes. By mapping these potential differences at single-nucleus resolution, we aim to identify novel, sex-specific molecular pathways that contribute to MDD pathophysiology, paving the way for more targeted therapeutic strategies.

## Materials and methods

### Animals

Three-month-old female and male wild-type C57BL/6 J mice obtained from Jiangsu Huachuang Xinuo Medical Technology Co., LTD were used for the experiments. The mice were divided into four groups and housed in a controlled environmental setting (temperature: 23 ± 2 °C, humidity: 50–60%, 12-hour light/dark cycle). All groups of mice had free access to food and water. This project was approved by Ethics Committee of experimental animal medicine of Southeast University (Serial No. 202362437).

The sample size was determined a priori through power analysis based on pilot data indicating an expected effect size (Cohen’s d) of ~1.3 for the primary outcome, which yielded a minimum requirement of n = 9 per group to achieve 80% power at α = 0.05; consequently, n = 10 mice per group were included to account for potential attrition. Following acclimation, all mice were randomly allocated to experimental groups using a computer-generated sequence by an investigator not involved in subsequent procedures to ensure unbiased group assignment. Pre-established exclusion criteria—defined prior to experimentation—included severe unrelated health issues, procedural fatalities, or statistical outliers identified by Grubb’s test (α = 0.05); based on these criteria, (~ 10%) animals were excluded from the final analysis.

### Sub-chronic variable stress (SCVS)

The Sub-chronic Variable Stress paradigm was employed based on its established efficacy in rapidly inducing robust anhedonia and anxiety-like behaviors, with a pronounced female bias, as previously validated [34]. This model, characterized by its short duration and alternating stressors to prevent habituation, is particularly suited for investigating early, sex-specific molecular adaptations to stress. Mice were housed in groups of 3 to 5 in a cage and allowed to acclimatize for one week before the experiments. After that, they will be exposed to three different stressors, alternating between them over a period of six days to avoid habituation. Stressors were administered in the following order: one hundred 0.45 mA/s foot-shocks delivered at random intervals for 1 h; tail suspension stress for 1 h; place in a perforated tube for 1 h within their home-cage. The above steps will be carried out continuously for three days, followed by a repetition of the same procedures for another three days.

### Behavioral tests

The assessment of depressive-like and anxiety-like behaviors in mice will begin one day after the completion of SCVS. Before behavioral testing, the mice will be acclimated to the testing room for 1 to 2 h. The behavioral tests will then be conducted in the following order:

### Open field test (OFT)

A white box (40 ×40 × 40 cm) was placed in a soundproof room illuminated with red light (80–100 lux). The mice were then placed in one corner of the white box and allowed to explore for 5 min. The total distance traveled by the mice, as well as the distance and time spent in the central area measuring 20 × 20 cm, were tracked and analyzed using DigBehv software.

### Forced swim test (FST)

A transparent cylindrical container with a capacity of 5 liters was placed in a room at a temperature of 24–25 °C, and 3.5 liters of water was then added. Subsequently, the mice were placed in the cylindrical container, and their behavior was recorded for 6 min, during which the duration of immobility was observed. Immobility time, defined as passive floating with only minimal movements necessary to keep the head above water, was scored during the last 4 min of the 6-minute test.This procedure is standard practice, as it allows mice to transition from initial escape-oriented behavior to a state of passive coping, which is considered to model behavioral despair [35]. The experiment was conducted by an observer who was blind to the experimental groups, and scoring was performed during the last 4 min of the recording.

### Novelty suppressed feeding (NSF)

After a 24-hour fasting period, the mice were placed in a corner of a white plastic box (40 ×40 × 40 cm), with a piece of mouse food placed in the center. The box was enclosed in a soundproof room illuminated with red light (80–100 lux). During the 10-minute test, the latency to feeding was measured by an observer who was blind to the experimental groups. Following the test, the food pellet was retrieved and weighed to quantify the amount consumed during the test session.

### Sucrose preference test (SPT)

A 0.5% sucrose solution was used, as this concentration is well-established in the literature to be highly palatable to C57BL/6 mice while avoiding potential ceiling effects in consumption that can occur with higher concentrations. Both sucrose and water intake were normalized to the body weight of each mouse (mL/g) for all analyses. Each mouse was individually housed in a cage equipped with two water bottles, allowing them to feed freely. On day 1, the mice were allowed to acclimate to the environment with free access to water for 24 h. From days 2 to 4, one bottle of regular drinking water in each mouse’s cage was replaced with a 0.5% sucrose solution. In the following days, the positions of the bottles were swapped daily to minimize the potential for positional preference among the mice. The amount of water and sucrose solution consumed was recorded each day. On day 5, the remaining volumes of water and the 0.5% sucrose solution were recorded. At the end of the testing period, all animals were returned to group housing.

### Measurement of serum corticosterone and 5-HT levels

Immediately after completing the behavioral measurements, blood collection was performed from the retro-orbital sinus of mice briefly anesthetized with isoflurane. Approximately 100 μL of whole blood was collected into a 0.5 mL tube and allowed to clot at room temperature for 1 h. The sample was then centrifuged at 1200 rpm for 15 min, and the supernatant was collected as serum, which was stored at −80 °C for later analysis. The concentrations of serum corticosterone and 5-HT were determined using ELISA kits from Abcam (Catalog #: ab108821 for corticosterone and Catalog #: ab133053 for 5-HT), following the manufacturer’s instructions for the experimental procedures.

### Single nucleus isolation and library preparation

Hippocampal tissues used for snRNA-seq were collected from an independent cohort of mice that did not undergo behavioral testing or blood collection, to preclude the confounding effects of cumulative stress on the transcriptome. The hippocampal tissues from the 3 mice within each experimental group were physically pooled together prior to the single-nucleus dissociation procedure to create one biological sample per group. The preparation of the single-nucleus suspension was then carried out as described in the previous study [36]. Briefly, freshly dissected hippocampi from each group were washed twice with cold RPMI 1640 medium containing 0.04% BSA. The tissues were then chopped into 1 mm pieces and digested into single cells using Neural Tissue Dissociation Kit (Cat. No. 130-092-628, Miltenyi Biotec, Bergisch Gladbach, North Rhine-Westphalia, Germany). Single-nucleus suspension was filtrated by BD FALCON 40 μm cell filter (BD, Franklin Lakes, NJ, USA), then centrifuged 300 g at 4 °C for 5 min. The supernatant was treated with 500 μL of culture medium and 500 μL of red blood cell lysis buffer solution (Cat. No. 130-094-183, Miltenyi Biotec) at 4 °C for 10 min. Then, it was centrifuged and washed twice, and finally resuspended in 100 μL of culture medium. The cell concentration and viability of the tissue suspension were assessed using the Luna II™ Automated Cell Counter (Logos Biosystems, Anyang-si, Gyeonggi-do, South Korea). Cell suspension with 1000 living cell/μL was used to construct library by Chromium Next GEM Single Cell 3ʹ Reagent Kits v3.1 (Cat. No.1000268, 10 x Genomics, Pleasanton, CA, USA), and sequenced via Illumina Nova 6000 PE150 platform. Detailed sequencing and quality metrics for each individual sample are provided in Supplementary Table S1.

To minimize technical variation, nuclei isolation, library preparation, and sequencing for all samples from the four experimental groups were performed simultaneously using the same batch of reagents and by the same operator. Consequently, no batch effect correction was applied to the dataset, as all samples were processed in a single batch.

### Integrated snRNA-seq processing and functional annotation

Raw sequencing data (FASTQ format) were processed through the Cell Ranger software suite (10x Genomics) using the mkfastq and count commands for demultiplexing, barcode processing, alignment, and gene expression UMI counting. Subsequent quality control and analyses were performed using the Seurat R package. Low-quality cells and potential doublets were removed using the following criteria: 1) cells with fewer than 200 detected genes were excluded; 2) cells with mitochondrial gene content exceeding 10% were excluded; and 3) doublets were identified and removed using Scrublet. After rigorous quality control, a total of 31,256 high-quality cells were retained.

Downstream analysis included data normalization using the LogNormalize method, selection of highly variable genes, principal component analysis (PCA), and cell clustering based on the Louvain algorithm. Dimensionality reduction and data visualization were accomplished using the UMAP algorithm. Identification of differentially expressed genes (DEGs) between experimental groups was conducted using the Wilcoxon rank-sum test, with an adjusted *p*-value < 0.05 considered significant. Functional enrichment analysis was performed using the Gene Ontology (GO) and Kyoto Encyclopedia of Genes and Genomes (KEGG) databases via the ClusterProfiler R package. For each cell type, differentially expressed genes (DEGs, defined as FDR < 0.05 and |logFC | > 0.25) were tested for over-representation against the background set of all genes expressed in that cell type. Enrichment terms with adjusted *p* < 0.05 (Benjamini-Hochberg) were considered statistically significant.

### Transcriptomics differential expression analysis

We performed differential gene expression analyses for multiple cell types with established involvement in depression pathology, including excitatory neurons, inhibitory neurons, oligodendrocytes, microglia, and astrocytes. Specifically, employing the Poisson generalized linear model implemented in the FindMarkers function of the Seurat package, we identified differentially expressed genes (DEGs) between SCVS-treated and untreated controls. The Benjamini-Hochberg method was applied to correct *p*-values for multiple hypothesis testing. Genes meeting both criteria (adjusted *p*-value < 0.05 and |logFC| > 0.25) were defined as significantly differentially expressed (DEGs) and assigned to the following categories: 1. Sex-specific DEGs: Genes exhibiting significant differential expression (adjusted *P* < 0.05) in exclusively one sex, with the other sex showing no significant change (nominal *P* > 0.1). This strict non-significance threshold reduces misclassification risk. Interaction analysis was avoided due to higher power requirements and potential for increased false positives under high logFC variance; 2. Sex-dimorphic DEGs: Genes exhibiting significant expression changes (FDR < 0.05) in females and males, but with log fold changes of opposing signs.

### Statistical analysis

Statistical analysis was conducted with GraphPad Prism version 8.2 (GraphPad Software, Inc., USA) or SPSS Statistics 21 (IBM, Armonk, NY, USA). The Shapiro-Wilk test evaluated data for normal distribution. Two-group comparisons utilized the two-tailed Student’s t-test, unless assumptions of normality and homoscedasticity were violated, in which case the Wilcoxon-Mann-Whitney test was employed. For multiple group comparisons, one-way or two-way ANOVA was performed, with Tukey’s or Bonferroni’s post hoc tests applied where appropriate. Results are expressed as mean ± SEM.

## Results

### Female mice exhibited notable anhedonia after SCVS treatment

**Fig. 1:**
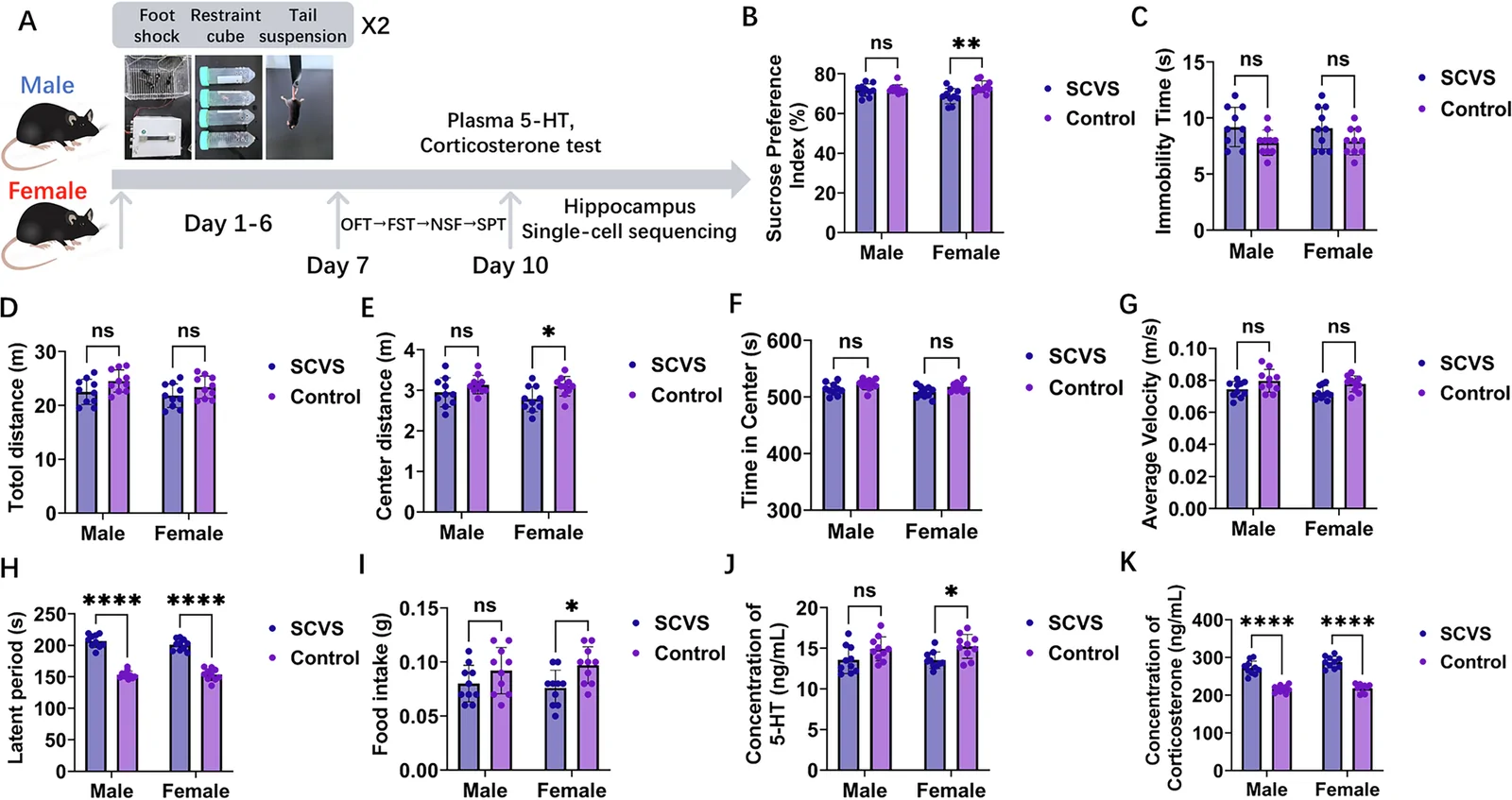
Construction of the SCVS model. **A** Flow chart of the sub-chronic variable stress (SCVS) model in male and female mice. **B** The percentage of sucrose preference in the SPT. **C** Immobility time in the FST. **D** OFT showed total distance. **E** Center distance. **F** Time in center and **G** average velocity in the OFT. **H** Latent period in the NSF. **I** Food intake. **J** Plasma 5-HT levels. **K** Serum corticosterone level. Data are represented as mean ± SEM. n = 10 mice per group. Statistical significance was determined by two-way ANOVA followed by post-hoc tests. *p < 0.05, **p < 0.01, ***p < 0.001, ****p < 0.0001.

To evaluate the behavioral effects of sub-chronic variable stress (SCVS), we randomly assigned twenty male and twenty female C57BL/6 J mice to either control or SCVS-treated groups (i.e., Male-Control n = 10, Male-SCVS n = 10; Female-Control n = 10, Female-SCVS n = 10). As illustrated in Fig. 1A, mice were subjected to SCVS paradigm followed by a battery of behavioral tests. The investigator was blinded during the experiment and data assessment.

The behavioral examination had been used to evaluate depressive-like behaviors in mice. Consistent with prior study demonstrating heightened stress sensitivity in females [34], a two-way ANOVA of the sucrose preference test revealed a significant main effect of treatment (SCVS) (F (1, 18) = 5.74, p = 0.027) and a significant treatment × sex interaction (F (1, 18) = 6.46, p = 0.020). The main effect of sex was not significant (F (1, 18) = 1.19, p = 0.289). Subsequent post-hoc multiple comparisons further confirmed that although both sexes showed a reduction in sucrose intake, SCVS treatment led to a significantly more pronounced decrease in female mice compared to males (Fig. 1B). In the forced swim test, two-way ANOVA revealed that treatment was a significant factor affecting immobility time (F (1, 18) = 5.42, p = 0.031). However, neither the main effect of sex (F (1, 18) = 0.00, p = 0.999) nor the treatment × sex interaction (F (1, 18) = 0.067, p = 0.798) reached statistical significance. Subsequent post-hoc multiple comparisons further confirmed that while SCVS exposure generally induced behavioral despair at the overall level, this effect was not specific to either sex and was not strong enough to reach statistical significance in any specific inter-group comparisons (Fig. 1C). It was determined by two-way ANOVA that no statistically significant main effects of treatment or sex, nor any interaction between these factors, were present for the measures of total distance traveled, average velocity, or time spent in the center of the open field (Fig. 1D, F, G). This supports the conclusion that baseline locomotor activity and center exploration were similar between groups. For center distance (Fig. 1E), the analysis indicated a significant main effect of treatment (F (1, 18) = 8.912, p = 0.008). Post-hoc tests confirmed that this measure was specifically reduced in SCVS-treated female mice. In the novelty suppressed feeding (NSF) test, two-way ANOVA revealed a significant main effect of treatment on the latency to feed (F (1, 18) = 315.6, p < 0.0001), without a significant main effect of sex or a treatment × sex interaction. Post-hoc analysis confirmed that both male and female SCVS-treated mice exhibited a significant prolongation of feeding latency compared to their respective control groups (Fig. 1H), indicating heightened anxiety-like behavior in response to stress. For food consumption during the test, a significant main effect of treatment was solely observed (F (1, 18) = 15.48, p = 0.001), with no other factors reaching statistical significance. Subsequent post-hoc tests identified that only female SCVS mice consumed significantly less food than female controls (Fig. 1I), suggesting a broader stress-induced reduction in motivational drive or appetite that was specific to females.

In addition, two-way ANOVA of plasma serotonin (5-HT) levels (Fig. 1J) revealed a significant main effect of treatment (F (1, 18) = 10.43, p = 0.005). Post-hoc analysis confirmed that sub-chronic variable stress (SCVS) exposure led to a significant reduction in plasma 5-HT levels in female mice, with no statistically significant change observed in males. Conversely, analysis of serum corticosterone (Fig. 1K) showed a significant main effect of treatment (F (1, 18) = 224.1, p < 0.0001), without a significant main effect of sex or an interaction effect. This indicates that SCVS robustly increased corticosterone levels in both male and female mice. We also monitored the impact of stress on body weight. SCVS treatment resulted in a significant attenuation of body weight gain in female mice, whereas no significant difference was observed in males. To account for baseline differences in body size, all fluid intake values (sucrose and water) were normalized to individual body weight (mL/g) prior to analysis.

Based on the test results, it was observed that female mice treated with SCVS exhibited more significant differences in the sucrose preference test and the novelty-suppressed feeding test. This suggests that SCVS treatment induced anxiety- and depression-like behaviors in female mice, accompanied by reduced plasma serotonin levels.

### Single-nucleus atlas of the hippocampus in mice with SCVS

**Fig. 2:**
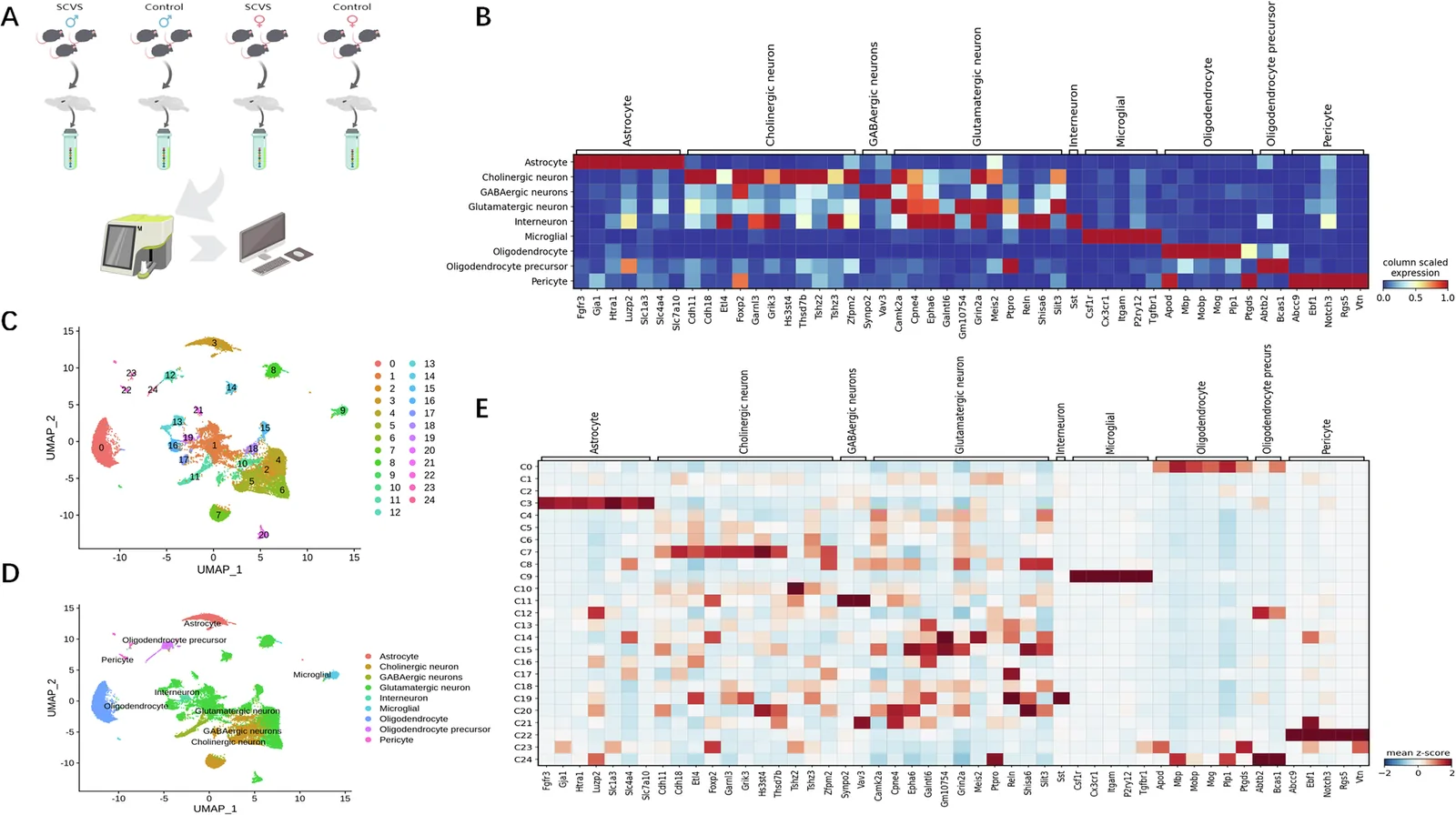
Identification of cell types. **A** Schematic representation of the experimental procedures (n = 3 mice per group). **B** Dotplot of representative markers for each cell types. **C, D** UMAP visualization showed clustering of 31,256 nuclei after removing doublets, colored by unsupervised cluster (C) and annotated cell types (D). **E** Dotplot of representative markers for each cell clusters.

To investigate the hippocampal transcriptional response to stress at single-nucleus resolution, we performed single-nucleus RNA sequencing (snRNA-seq) on an independent cohort of mice (n = 3 per group) using the 10x Genomics platform. This analysis encompassed the four experimental groups: Male-Control, Male-SCVS, Female-Control, and Female-SCVS (Fig. 2A). Each set of data contained three biological replicates. High-quality transcriptome data were obtained from each sample after quality control analysis. A total of 31,256 cells were analyzed across four cohorts: female controls (n = 8512), female SCVs (n = 8597), male controls (n = 7183), and male SCVs (n = 6964). Single-nucleus transcriptomic quantification revealed distinct per-cell molecular outputs across groups. Female control cells exhibited medians of 1064 genes and 16,796 transcripts, while female SCVS cells showed 1222 genes and 21,135 transcripts. Male control cells displayed 1025 genes and 24,113 transcripts, compared to 1718 genes and 29,739 transcripts in male SCVS cells (Table S1). Gene and transcript counts demonstrated comparable distributions across experimental groups. There were no statistically significant differences in the number of genes or transcript counts between the groups (Figure S1). Based on “GENCODE” Dataset and well-acknowledged biomarkers (Fig. 2B, Table S2), we have collected the cell types contained in the sequencing samples, as well as the typical cell marker genes for each cell type. To assess cellular heterogeneity, we conducted dimensionality reduction analysis using uniform manifold approximation and projection (UMAP), generating two-dimensional embeddings of the dataset. We have defined 25 clusters in all samples (Fig. 2C), which were further divided into 9 cell populations as follow: Astrocyte, Cholinergic neurons, Glutamatergic neurons, GABAergic (non-specific) neurons, Sst-positive interneuron, Microglia, Oligodendrocyte, Oligodendrocyte precursor, Pericyte (Fig. 2D).

As shown in Fig. 2E and Supplementary Figures S2–S9, Cluster 0 was identified as oligodendrocytes, based on the high expression of canonical marker genes such as Mbp, Mobp, Mog, and Plp1. Cluster 12 and 24 robustly expressed Abtb2 and Bcas1 were oligodendrocyte precursor cells. Cluster 22 and 23 were considered as pericyte by the hallmarks of Ebf1 and Vtn. In addition, Cluster 22 displaying a high expression of Abcc9, Notch3 and Rgs5. Cluster 3 had many specific biomarkers (Fgfr3, Gja1, Htra1, Luzp2, Slc1a3, Slc4a4 and Slc7a10), which were defined as astrocytes. Cluster 5, 7 and 10 could be considered as cholinergic neurons with expression of Cdh11, Cdh18, Etl4, Garnl3, Grik3, Hs3st4, Thsd7b and Tshz3. In particular, Cluster 10 was additionally expressed Tshz2. Cluster 2 and 11 displaying a high expression of Sympo2 and Vav3 suggested GABAergic (non-specific) neurons. Glutamatergic neurons have many specific markers, as cell clusters 1, 4, 8, 13, 14, 15, 16, 17, 18, 20, and 21 each contain various different specific markers for glutamatergic neurons, indicating a glutamatergic neuron population more likely. Cluster 19 was considered as Sst-positive interneuron with high expression of Sst. Cluster 9 had their own specific biomarkers of microglia (Csf1r, Cx3cr1, Itgam, P2ry12 and Tgfbr1). In summary, based on our analysis of various classic biomarkers in hippocampus, we have summarized nine types of acknowledged cell populations.

### The transcriptional response to SCVS is sex and cell type specific

**Fig. 3:**
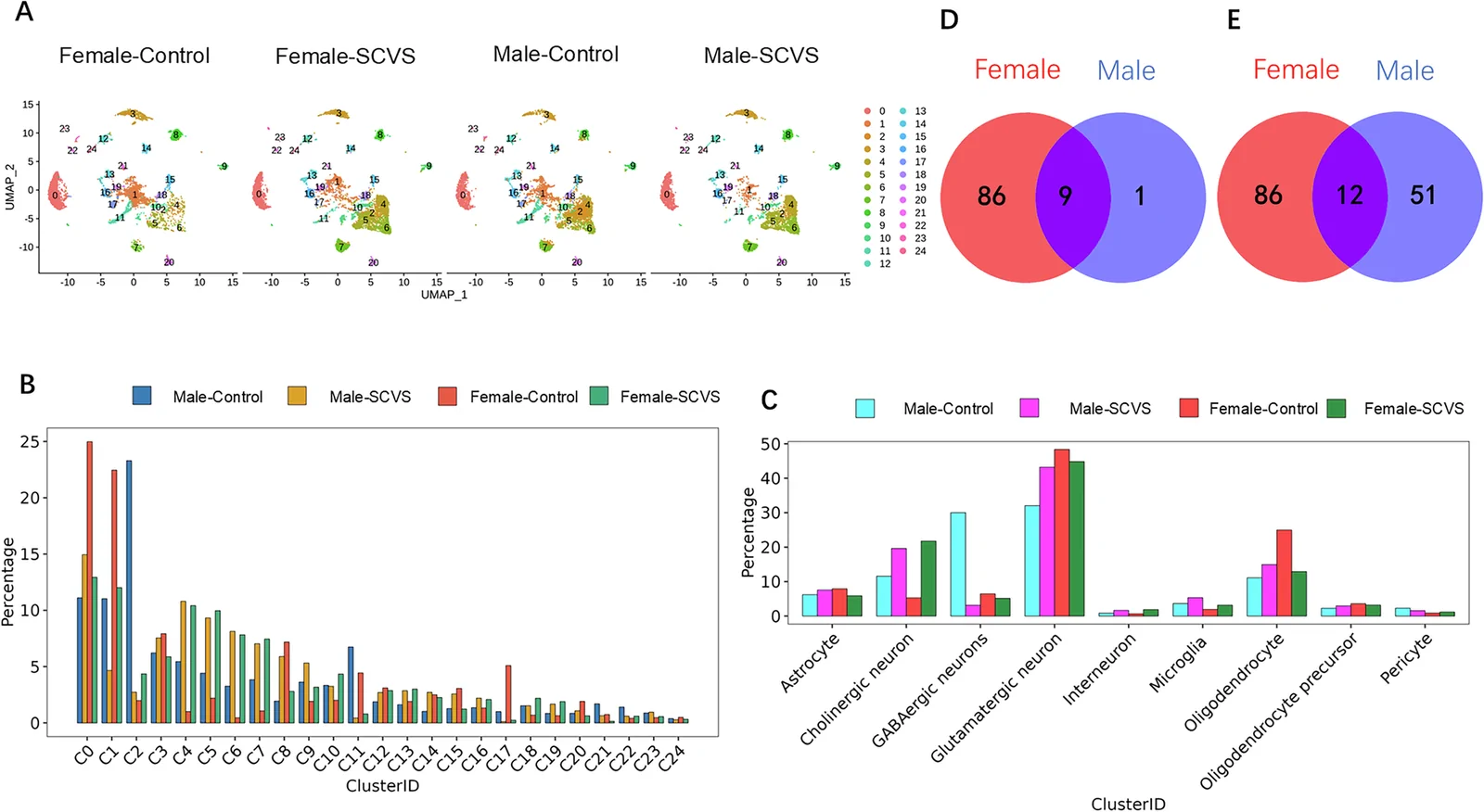
Differential gene expression between male and female mice treated with SCVS. **A** The UMAP showed the uniform distribution of nuclear clusters in two groups. **B** Fraction of each cell cluster from different samples. **C** Fraction of each cell type from different samples. **D, E** Venn diagrams showing upregulated and downregulated DEGs in male vs. female mice.

Although depression exhibits a well-documented and persistent female predominance in prevalence, the biological mechanisms driving this sex disparity, particularly at the cellular level, remain incompletely understood. To elucidate the biological mechanisms underlying sex-specific differences in depression risk, cell type counts in males and females were analyzed following SCVS treatment. By counting each cell cluster in the hippocampus of the four groups (Fig. 3A), compared with the SCVS treatment group, the female-control group had a large number of oligodendrocytes (Cluster 0), while the male-control group had more abundant GABAergic (non-specific) neurons (Cluster 2,11) (Fig. 3B, C). Importantly, while the calculated proportions of individual cell populations may not precisely mirror their true distribution within the mouse brain, statistical analysis of the cellular composition revealed significant alterations. Specifically, SCVS exposure led to a significant reduction in the proportion of oligodendrocytes in females, and a shift in GABAergic neurons between sexes. These dynamic changes in cellular composition, particularly in oligodendrocytes, may contribute to the modulation of anhedonic behaviors in females.

To better understand the role of sex in depression progression, we analyzed the extent of transcriptional alterations in the hippocampus following SCVS exposure. The Wilcoxon rank-sum test was used to identify differentially expressed genes (DEGs) between SCVS-treated and control groups for each cell type. To quantify the overall transcriptional response, we aggregated DEGs across all cell types identified in the hippocampal snRNA-seq dataset. Strikingly, female mice exhibited three times more differentially expressed genes (DEGs) than males. Specifically, we identified 86 female-specific upregulated DEGs, contrasting with only one male-specific upregulated DEG and minimal overlap of just 9 shared DEGs (Fig. 3D). In addition, there are 86 specific downregulated DEGs in females, 51 in males and a limited overlap of 12 DEGs (Fig. 3E). Astrocyte, cholinergic neurons, GABAergic (non-specific) neurons and glutamatergic neurons exhibited the highest number of DEGs, while Sst-positive interneuron, oligodendrocyte, microglia and pericyte exhibited few changes in DEGs in both sexes (Figure S10). In males, a high proportion of GABA neurons in cluster 2 expressed more upregulated DEGs than in females, while downregulated DEGs was much lower than in females. However, oligodendrocytes in cluster 0 not only highly expressed more upregulated DEGs, but also overexpressed more downregulated DEGs (Figure S11, S12).

**Fig. 4:**
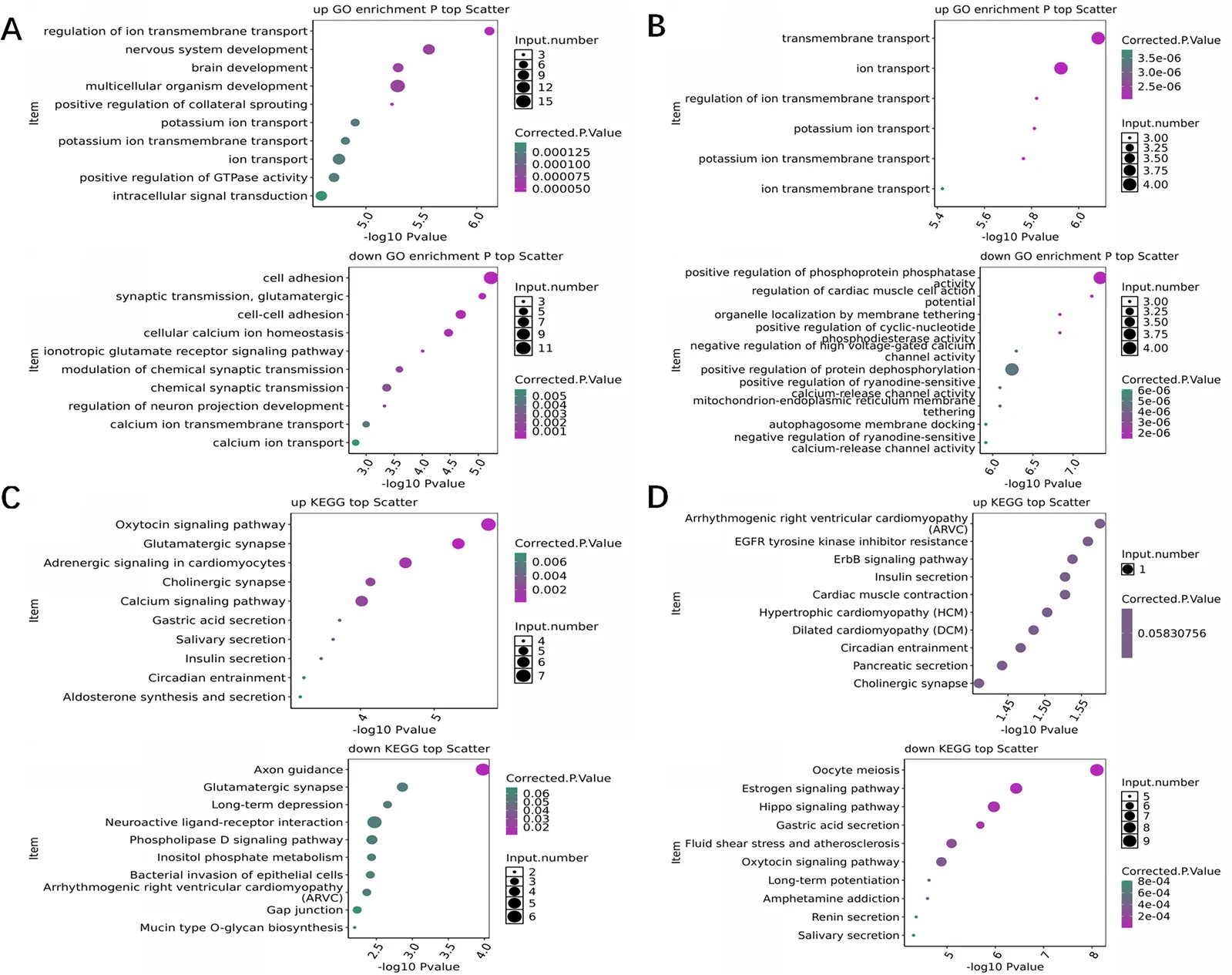
GO and KEGG Analysis Reveals Sex-Specific Pathway Enrichment after SCVS. **A** Female-Specific GO Enrichment of Differentially Expressed Genes (Top: Upregulated Pathways; Bottom: Downregulated Pathways). **B** Male-Specific GO Enrichment of Differentially Expressed Genes (Top: Upregulated Pathways; Bottom: Downregulated Pathways). **C** Female-Specific KEGG Pathway Enrichment of Differentially Expressed Genes (Top: Upregulated Pathways; Bottom: Downregulated Pathways). **D** Male-Specific KEGG Pathway Enrichment of Differentially Expressed Genes (Top: Upregulated Pathways; Bottom: Downregulated Pathways).

As illustrated in Fig. 4A, GO enrichment analysis revealed that in female subjects subjected to SCVS, a significant proportion of upregulated genes were associated with key biological processes. These included “Regulation of ion transmembrane transport”, “Nervous system development”, “Brain development” and “Multicellular organism development”. Conversely, genes exhibiting downregulation were predominantly enriched in functional categories related to “Cell adhesion”, “Cell-cell adhesion” and “Synaptic transmission”. In stark contrast, the transcriptional response in SCVS-treated males displayed a markedly different pattern (Fig. 4B). Here, both upregulated and downregulated genes showed significantly less diverse functional enrichment. The primary GO terms identified were confined to “Transmembrane transport”, “Ion transport” and “Regulation of phosphoprotein phosphatase activity” indicating a more restricted molecular response compared to females. Intriguingly, KEGG pathway analysis further highlighted profound sex-specific differences in the global gene expression response to SCVS (Fig. 4C, D). A particularly notable finding was the significant dysregulation of genes within the “Oxytocin signaling pathway”. This pathway was predominantly altered in females, while in males, oxytocin signaling-related genes were specifically downregulated or suppressed. This distinct, sex-biased pathway modulation strongly suggests that SCVS elicits fundamentally divergent molecular adaptations in males and females, with oxytocin signaling emerging as a key pathway exhibiting sexual dimorphism in the stress response.

### Sex-biased cell type-specific gene expression

To investigate sex-specific transcriptional changes, we conducted cell type-specific differential gene expression analysis comparing SCVS mice with control cohorts using sex-stratified comparisons. Following rigorous multiple testing correction (Benjamini-Hochberg procedure, FDR < 0.05), we identified numerous significantly dysregulated genes exhibiting sex-dimorphic expression patterns across distinct cellular populations. The top 10 DEGs exhibiting male-specific, female-specific, and sexually dimorphic expression patterns are presented in Tables 1–3, respectively.

**Table 1:** Male-exclusive differentially expressed genes (DEGs) with the largest absolute log₂ fold change (|logFC|) in each cell type, comparing SCVS to controls.

Cell Type	Gene symbol	Min and max FDR significance
Astrocytes	UP:Esco1,Nrg1,Phactr1,Dcc,Gm28175,Epha5,Kcnq5,Gria2,Lrrtm4; DOWN:A330076C08Rik,Gm12239,Itpkb,Slc6a11,Mpp6,Lrmda,mt-Co1,Robo1,Gtf2ird1	4.66e-11—0.037
Cholinergic neurons	UP:1500004A13Rik,Hs3st5,Cdh18,Sdk1,Nrg1,Kctd16,Rbfox1,Pcsk2,Cdh9,Slc35f4; DOWN:Plp1,Adarb2,Norad,Hsp90ab1,Tmsb4x,Calm1,Dynll2,Aldoa,Ywhag,Calm3	2.22e-30—0.018
GABAergic neurons	UP:4930578G10Rik,Car10,Asic2,Fhod3,Csmd2,Gm12394,Sidt1,Fstl4,Kcnb1,Rbfox3; DOWN:Plp1,Tmsb4x,Tuba1b,Fth1,Uchl1,Norad,Prkcd,Rab3c,Spock3,Npas3	5.54e-22—0.010
Glutamatergic neurons	UP:Phactr1,Kcnh1,Hs6st3,Epha7,Kalrn,Dlgap2,Fstl4,Cracdl,Sphkap,Unc5d; DOWN:Plp1,Norad,Vav3,Rnf220,Dynll2,Tmsb4x,Calm1,Hsp90aa1,Ywhah,Hsp90ab1	5.83e-84—5.59e-13
Interneurons	UP:Dlgap1	0.002
Microglia	DOWN:Ntng1,Synpo2,Plekhg1,Nell1,Atp1a3,Kcnc2,Hsp90ab1,Lef1,Ywhag,Rcan2	6.43e-10—0.026
Oligodendrocytes	UP:Cnksr2,Dlgap2,Nrxn3,Csmd1,Dlg2,Lingo2,Lrrtm4; DOWN:Tcf7l2,Synpo2,mt-Nd3,Vav3,Rab3c,Slc17a6,Gnas,Kcnc2,Uchl1,Col25a1	2.22e-15—0.009
Oligodendrocyte precursor	UP:Nav3	0.0216
Pericytes	UP:Arhgap6,Cped1,Pid1,Maml2; DOWN:Ntng1,Kcnc2,Snap25,Plp1,Cntnap2	1.43e-06—0.040

*DEGs*, differentially expressed genes; *FDR*, false discovery rate; max, maximum; min, minimum.

**Table 2:** Female-exclusive differentially expressed genes (DEGs) with the largest absolute log₂ fold change (|logFC|) in each cell type, comparing SCVS to controls.

Cell Type	Gene symbol	Min and max FDR significance
Astrocytes	UP:Cspg5,St6galnac5,Ptn,Rgs20,Tspan7,Vegfa,Slco1c1,Meis2,Gfra1,Lypd6; DOWN:Pdgfd,Gria4,Ctnna3,Dpp6,Csmd3,Cables1,Ust,Meg3,St6galnac3,Aldh1a1	2.93e-48—7.37e-06
Cholinergic neurons	UP:Kcnt2,Mef2c,Zeb2,Garnl3,Slc17a7,Arhgap10,Lmo4,Dpp10,Satb2,Phf24; DOWN:Dpp6,Sgcd,Erbb4,Cntnap5b,Vwc2l,Zfhx4,Inpp4b,Shisa6,Rbms3,Cntnap5c	1.045e-80—2.21e-08
GABAergic neurons	UP:Mef2c,Zeb2,Chsy3,Gpm6b,R3hdm1,Arhgap10,Grm3,Dlg1,Nlk,Ext1; DOWN:Zfhx3,Rnf220,Ntng1,Rbms3,Grm1,Shisa6,Dpp6,Tshz2,Nxph1,Lhfpl3	5.90e-63—0.004
Glutamatergic neurons	UP:Mef2c,Dpp10,Meis2,R3hdm1,Satb2,Myo16,Rorb,Cpa6,Arpp21,Nlk; DOWN:Htr2c,Unc13c,Tenm1,Grid2,Nkain3,Cdh18,Zfp521,Zfpm2,Usp29,Olfm3	0—4.91e-43
Interneurons	UP:Unc13c,Satb1,Kcnip1,Cntn4; DOWN:Tenm3,C130073E24Rik,Unc5d,Sgcz,Gm45323,Greb1l,Cntnap5c,Tenm2,Lrrc4c	1.27e-08—0.026
Microglia	UP:Cst3,Pdzrn3,Dpp10,R3hdm1,Kcnq5,Kcnh7,Rsrp1,Nrg1,Phactr1,Ptprd; DOWN:Rmst,Htr2c,Gm32647,Trpm3,Fkbp5,Tenm1,Grid2,Plcl1	8.26e-17—0.022
Oligodendrocytes	UP:Mef2c,Pdzrn3,Satb2,R3hdm1,Ninj2,Plcb1,Pde1a,Meis2,Opalin,Sytl2; DOWN:Zbtb16,Sgk1,Adipor2,Nkain3,Htr2c,Trpm3,Dpp6,Galnt17,Enah,Usp29	2.44e-57—4.31e-06
Oligodendrocyte precursor	UP:Mef2c,Kcnh7,Vegfa,Cspg5,Chl1,Dpp10,Chst9,Ptprd,Cacna2d1,Kcnq5; DOWN:Rmst,Cdh8,Angpt1,Tmem117,Kazn,Cacnb2,Htr2c,Itga9,Usp29,Rit2	8.08e-22—0.043
Pericytes	UP:Dpp10,Celf2; DOWN:Zbtb16	1.16e-08—0.002

*DEGs*, differentially expressed genes; *FDR*, false discovery rate; max, maximum; min, minimum.

**Table 3:** Cell type-resolved sex-dimorphic DEGs with largest absolute log₂ fold change (|log₂FC|) between SCVS and controls.

		Male		Female	
Cell type	Gene symbol	avg_log2FC	FDR	avg_log2FC	FDR
Cholinergic neurons	Grid2	0.288004454857404	0.0490039038422343	-0.742521028	0.00446671677081214
Glutamatergic neurons	Grid3	0.458251432440163	1.25538411034689e-06	-0.633057624	3.81238459645348e-18
	Shisa6	0.409371997216913	1.6589071218597e-13	−1.032340986	1.28440917990769e-112
	Pip5k1b	0.331386436326284	1.65600575824378e-14	-0.673234507	2.74940136553801e-57
Oligodendrocyte	mt-Atp6	−0.483684928	1.42744656083428e-06	0.566210025116617	0.000548521862665919
	mt-Co3	−0.430521506	0.0169201109591201	0.602898331319292	0.00247733955070968

*DEGs*, differentially expressed genes; *FDR*, false discovery rate.

#### Male-specific differential gene expression

Male-specific DEGs exhibited distinct cell type–restricted patterns (Table 1). Neuregulin-1 (Nrg1) was markedly overexpressed in two cell types: astrocytes (FDR = 4.66e-11) and cholinergic neurons (FDR = 2.22e-30), with no significant changes in other populations. Nrg1 encodes a ligand for receptor tyrosine kinases and is critically involved in neurodevelopment and synaptic plasticity. In contrast, Slc6a11 showed male-specific downregulation restricted to astrocytes (FDR = 1.63e-05). Reduced Slc6a11 expression has been implicated in depression pathogenesis via impaired GABAergic transmission. Notably, while previous MDD literature has not reported sexually dimorphic expression of Plp1, our single-nucleus data revealed pronounced reductions of this gene specifically within cholinergic, GABAergic, and glutamatergic neuronal populations in the male hippocampus, highlighting a previously unrecognized cell type–specific sexual dimorphism.

#### Female-specific differential gene expression

Female-specific DEGs also exhibited striking cell type–specific patterns (Table 2). Vegfa was highly upregulated exclusively in astrocytes (FDR = 2.02e-20). Vegfa, together with Ptn, acts on the Ptprz1 receptor to regulate angiogenesis and cell migration, suggesting a potential role in stress-induced vascular remodeling. A particularly notable finding was the pan-cellular upregulation of Mef2c in females, which was observed across virtually all major hippocampal cell types: astrocytes (FDR = 6.69e-13), cholinergic neurons (FDR = 3.53e-52), GABAergic (non-specific) neurons (FDR = 2.26e-23), glutamatergic neurons (FDR = 0), oligodendrocytes (FDR = 2.44e-57), microglia (FDR = 7.41e-04), and oligodendrocyte precursor cells (FDR = 4.63e-06). This widespread upregulation suggests a conserved, female-specific transcriptional regulatory response to stress across diverse cell lineages.

#### Sex-dimorphic differential gene expression

Mitochondrial functional genes (such as those in the ATP synthase and COX families) show differential expression in depression. Oxidative stress-related analyses reveal that abnormal mitochondrial gene expression may exacerbate depressive symptoms through energy metabolism deficits [37, 38]. Genes encoded by mtDNA have long attracted scientific interest due to the maternal inheritance of mitochondria, and thus may contribute to research on sexual dimorphism [39]. Additionally, mtDNA is more susceptible to oxidative stress than nuclear DNA, which represents a major contributing factor to depression. Sexually dimorphic expression of mitochondrial DNA (mtDNA)-encoded genes was predominantly observed in oligodendrocytes (Table 3). Specifically, mt-Co2 and mt-Cytb showed opposing expression patterns: their expression increased in females following SCVS but decreased in males (FDR = 3.90e-05 and 1.24e-05, respectively). More strikingly, mt-Atp6 and mt-Co3 exhibited sexually antagonistic expression profiles: both genes were robustly upregulated in female oligodendrocytes but markedly downregulated in male oligodendrocytes after stress exposure. Given that mtDNA is maternally inherited and more susceptible to oxidative stress than nuclear DNA, these cell type–restricted, sex-dimorphic mitochondrial gene expression changes in oligodendrocytes may contribute to female-specific vulnerability to stress-induced metabolic dysregulation and oxidative damage. Since oxidative stress is a key component in the core pathophysiological network of depression, the more pronounced depression-like behaviors observed in female patients may be attributed to enhanced mitochondrial activity in oligodendrocytes, leading to increased levels of oxidative stress.

### Sex-stratified GO enrichment analysis across different cell types

To delineate biological pathways and processes underlying sex-specific expression changes in depression, we performed cell type–specific over-representation analysis using the Gene Ontology (GO) database. For each cell type, we tested male-specific and female-specific differentially expressed genes (DEGs) for enrichment against the background of all genes expressed in that cell type. This approach allowed us to identify depression-relevant biological processes exhibiting sex-dependent dysregulation across distinct cellular contexts.

#### Cell type–specific enrichment of male-specific upregulated genes

**Fig. 5:**
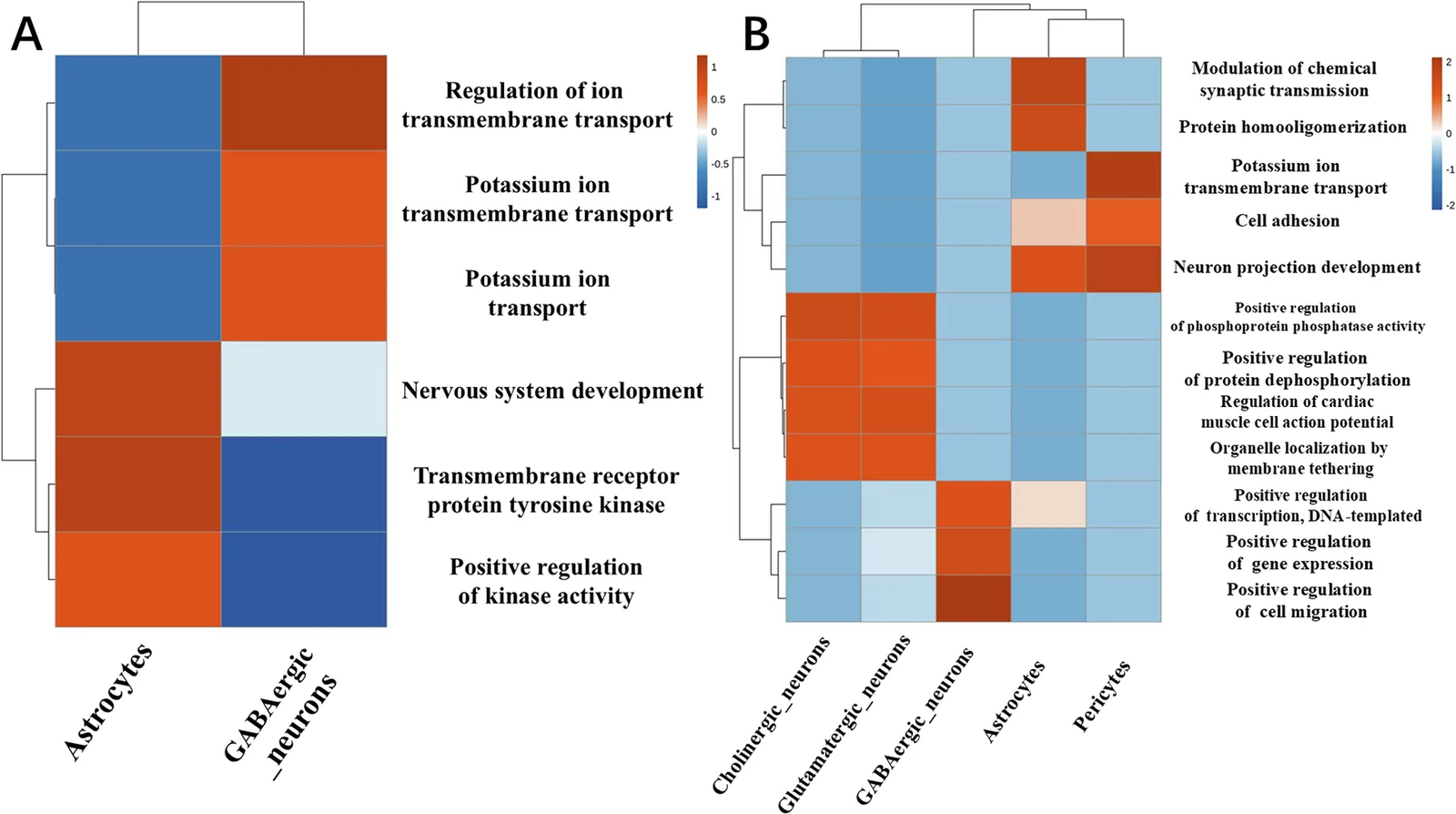
GO Enrichment Analysis of Male-Associated Biological Processes. **A** GO enrichment analysis of upregulated genes, stratified by cell type. **B** GO enrichment analysis of downregulated genes, stratified by cell type. The heatmaps display –log10(FDR) values, with darker shades indicating more statistically significant enrichment. Each row represents a specific cell type as indicated on the left, allowing direct visualization of cell type–specific pathway enrichment patterns.

Male-specific upregulated genes showed distinct, cell type–dependent functional enrichment patterns (Fig. 5A).

In astrocytes, upregulated genes were significantly enriched in pathways related to transmembrane receptor protein tyrosine kinase signaling (GO:0007169) and positive regulation of kinase activity (GO:0033674), suggesting enhanced growth factor signaling that may support neuroplasticity and cell survival. In GABAergic (non-specific) neurons, by contrast, upregulated genes were primarily enriched in potassium ion transmembrane transport (GO:0071805) and regulation of ion transmembrane transport (GO:0034765), pointing to a prioritized role in maintaining ionic homeostasis and controlling neuronal excitability. Both cell types shared enrichment in nervous system development (GO:0007399), indicating convergent contributions to hippocampal structural maturation despite their divergent specialized functions.

#### Cell type–specific enrichment of male-specific downregulated genes

Male-specific downregulated genes also exhibited clear cell type–restricted patterns (Fig. 5B).

In cholinergic and glutamatergic neurons, downregulated genes were enriched in positive regulation of phosphoprotein phosphatase activity (GO:1900182) and positive regulation of protein dephosphorylation (GO:0035305), suggesting impaired control over phosphorylation-dependent synaptic plasticity. In GABAergic (non-specific) neurons, downregulated genes were enriched in positive regulation of transcription, DNA-templated (GO:0045893) and positive regulation of gene expression (GO:0010628), along with neuron projection development (GO:0031175) and cell adhesion (GO:0007155), indicating reduced capacity for activity-dependent transcriptional responses and structural remodeling. In astrocytes, downregulated genes were enriched in potassium ion transmembrane transport (GO:0071805), pointing to compromised K⁺ buffering capacity that may disrupt extracellular ionic homeostasis.

#### Cell type–specific enrichment of female-specific upregulated genes

**Fig. 6:**
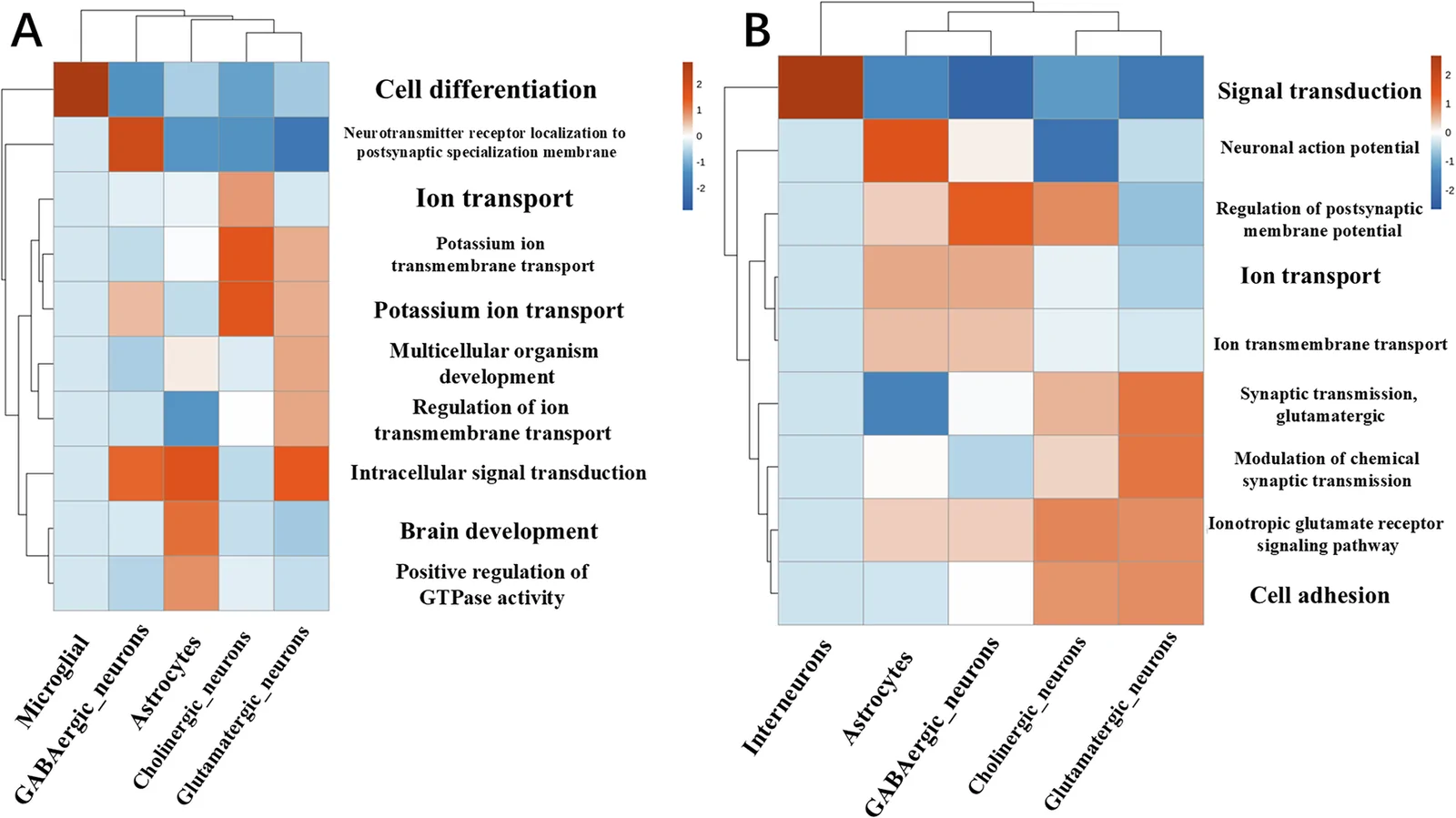
GO Enrichment Analysis of Female-Associated Biological Processes. **A** GO enrichment analysis of upregulated genes, stratified by cell type. **B** GO enrichment analysis of downregulated genes, stratified by cell type. The heatmaps display –log10(FDR) values, with darker shades indicating more statistically significant enrichment. Each row represents a specific cell type as indicated on the left, highlighting the cell type–restricted nature of the female stress response.

Female-specific upregulated genes revealed specialized functional programs across cell types (Fig. 6A).

In GABAergic (non-specific) neurons, upregulated genes were strongly enriched in neurotransmitter receptor localization to postsynaptic specialization membrane (GO:0099072), highlighting a female-specific emphasis on precise synaptic targeting and inhibitory circuit refinement. In astrocytes and multiple neuron clusters, upregulated genes were enriched in potassium ion transmembrane transport (GO:0071805) and related ion transport terms, suggesting coordinated deployment of astrocytic and neuronal K⁺ handling mechanisms to stabilize local microenvironments. Enrichment of positive regulation of GTPase activity (GO:0043547) across several clusters pointed to Rho/Ras-mediated cytoskeletal remodeling as a driver of synaptic organization in females.

#### Cell type–specific enrichment of female-specific downregulated genes

Female-specific downregulated genes revealed a coordinated suppression strategy targeting glutamatergic signaling and electrophysiological stability (Fig. 6B).

In glutamatergic neurons, downregulated genes were enriched in synaptic transmission, glutamatergic (GO:0035249) and ionotropic glutamate receptor signaling pathway (GO:0035235), indicating direct dampening of excitatory neurotransmission. In cholinergic neurons, downregulated genes were enriched in neuronal action potential (GO:0019228), while in SST-positive interneurons and astrocytes, regulation of postsynaptic membrane potential (GO:0060078) was suppressed, collectively constraining both synaptic input integration and neuronal output firing. In glutamatergic and other neuron clusters, reduced cell adhesion (GO:0007155) suggested impaired structural rewiring. Collectively, these downregulated genes act on dual axes in females: directly inhibiting glutamatergic excitatory drive and broadly constraining electrophysiological dynamics, shaping a network-level suppression of hippocampal excitability and plasticity with cell type–differentiated roles.

## Discussion

This study established a comprehensive single-nucleus atlas of the hippocampal response to sub-chronic variable stress (SCVS), revealing profound sexual dimorphism at the molecular level. We observed divergent behavioral phenotypes, with female mice exhibiting a more pronounced reduction in measures such as sucrose preference, accompanied by a selective decrease in plasma serotonin (5-HT) levels. The specific reduction in female plasma 5-HT levels, while not a direct proxy for central activity, has been reported in some clinical studies of MDD, though its significance and reliability remain debated [40]. It is crucial to emphasize that peripheral 5-HT originates predominantly from the gut and platelets and does not cross the blood-brain barrier [41]. Therefore, this finding in our model may reflect a systemic, sex-specific stress response rather than a direct mechanism for central anhedonia. Additionally, it is crucial that these phenotypic differences are associated with fundamentally distinct transcriptional reprogramming in the hippocampus. Female mice mounted a much broader transcriptional response, with a three-fold greater number of differentially expressed genes than males and minimal overlap. This striking molecular divergence suggests that males and females engage disparate biological pathways in response to the same stressor, a finding that substantially advances our understanding of sex differences in stress pathophysiology.

Crucially, these behavioral phenotypes correlate with profound sex-specific cellular reorganizations within the hippocampus. Female controls exhibited a baseline oligodendrocyte predominance that was substantially diminished post-stress, a finding with direct implications for myelination dynamics. Oligodendrocytes, responsible for axonal myelination and metabolic support, play critical roles in maintaining neural circuit integrity and signal conduction velocity [42]. The stress-induced depletion of this population in females may therefore disrupt hippocampal network synchronization, particularly affecting regions involved in reward processing and emotional regulation [20, 43]. Our finding of stress-induced depletion of the oligodendrocyte population in females aligns with postmortem evidence from MDD patients, which demonstrates reduced expression of oligodendrocyte-specific genes and ultrastructural myelin deficits [44]. Concurrently, we observed female-specific dysregulation of maternally inherited mitochondrial DNA (mtDNA)-encoded genes-including mt-Atp6 and mt-Co3 upregulation-specifically within oligodendrocytes. This finding strongly suggests that mitochondrial dysfunction in oligodendrocytes may be a core mechanism of depression in females, suggesting that a higher sensitivity to oxidative stress could impair both myelination and the metabolic support essential for maintaining synaptic plasticity.

The transcriptional landscapes revealed fundamentally divergent adaptive strategies between sexes. Females displayed threefold more differentially expressed genes (DEGs) than males with minimal overlap (9 shared DEGs), underscoring distinct pathophysiological trajectories rather than mere quantitative variations. Female-specific signatures included astrocytic Vegfa upregulation, which may drive neuroinflammatory and vascular pathology through interactions with the Ptprz1 receptor, as previously implicated in depression-related angiogenesis [45, 46]. Additionally, the phenomenon of a widespread increase in Mef2c expression across all major hippocampal cell types in female individuals highlights a conserved regulatory response. However, this phenomenon contradicts the previous article that described a male-specific expression of Mef2c in neurodevelopment and immune responses [47]. Thus, whether Mef2c serves as a potential regulator for female-specific stress adaptation remains controversial.

Notably, the oxytocin signaling pathway was selectively suppressed in stressed females, providing a molecular correlate for impaired social reward processing-a core feature of depression with known sex disparities [48, 49]. Oxytocin, critical for social bonding and reward, may represent a key node in the female-specific stress response network [48]. Conversely, males exhibited perturbations centered on synaptic signaling fidelity: NRG-1 overexpression in astrocytes and cholinergic neurons may disrupt neurotrophic support, as NRG-1 has been linked to impaired synaptic plasticity in depression [50]. Meanwhile, Slc6a11 downregulation in astrocytes could compromise GABAergic transmission, potentially contributing to excitatory-inhibitory imbalance [51]. The sex-antagonistic expression profiles of mtDNA genes in oligodendrocytes (mt-Co2 and mt-Cytb upregulated in females, downregulated in males) further establish mitochondrial function as a key determinant of sex-specific stress vulnerability, with implications for energy metabolism and reactive oxygen species generation.

Our data also reveal a pronounced, male-specific reduction in PLP1 expression within cholinergic, GABAergic, and glutamatergic neuronal populations, a finding that appears to contrast with some existing literature which reports no sexually dimorphic expression for this gene in MDD. This discrepancy may be explained by two key factors. First, technical resolution: previous bulk-tissue analyses would average signals across all cell types, potentially masking the cell type-specific downregulation we observed at single-nucleus resolution, as the strong expression of PLP1 in oligodendrocytes could overshadow subtler changes in neurons. Second, context specificity: the sexually dimorphic expression of PLP1 may be uniquely unmasked by the sub-chronic variable stress (SCVS) paradigm, highlighting the importance of stressor type and timing. Regarding its relevance to MDD, this finding suggests a role for PLP1 that extends beyond its canonical function in myelination. Its specific downregulation in male cholinergic neurons points to a potential mechanism involving impaired cholinergic signaling, a system critically involved in cognition and mood regulation. This aligns with the concept of sex-divergent adaptive strategies, where males, in response to stress, may undergo more refined adjustments in specific neurotransmitter systems and myelin-related genes like PLP1, potentially leading to a different set of functional impairments compared to the broader transcriptomic and glial metabolic alterations observed in females.

Pathway analyses delineated distinct circuit-level consequences. Female hippocampi demonstrated coordinated suppression of glutamatergic synaptic transmission-particularly through ionotropic glutamate receptor pathways-alongside impaired K⁺ buffering in astrocytes and reduced cell adhesion molecules. This unified suppression of excitatory drive, ionic homeostasis, and structural plasticity aligns mechanistically with observed depressive-like behaviors, as glutamatergic signaling is central to reward processing and synaptic plasticity [52]. Males, however, exhibited dysregulation focused on signaling efficiency rather than broad excitability changes: diminished phosphoprotein phosphatase activity in glutamatergic neurons suggests dysregulated phosphorylation-dependent synaptic plasticity, while transmembrane transport alterations indicate compromised neuronal communication fidelity. These differences may explain why males showed less severe depressive phenotypes, maintaining some capacity for synaptic adaptation despite stress exposure.

The female-specific enrichment of “neurotransmitter receptor localization to postsynaptic specialization” in GABAergic (non-specific) neurons further implies that precision in synaptic targeting is selectively compromised in females. This finding aligns with our observation of reduced cell adhesion molecules, suggesting a multifaceted disruption of synaptic architecture. Together, these changes would impair the fine-tuning of inhibitory control, potentially exacerbating excitatory drive in key circuits. In contrast, male GABAergic (non-specific) neurons prioritized potassium ion transmembrane transport, reflecting a focus on maintaining ionic homeostasis-a strategy that may preserve basic neuronal function at the expense of plastic adaptations [53].

These findings substantially advance depression pathophysiology models in three critical dimensions. First, they shift focus beyond neuronal-centric paradigms by establishing oligodendrocytes-through their sexually dimorphic mitochondrial genomics-as key contributors to female vulnerability. This challenges the traditional emphasis on neurons and microglia in depression research, highlighting the need for glial-centric therapeutic approaches. Second, they resolve longstanding controversies regarding sex differences by demonstrating that male and female stress adaptations involve fundamentally distinct molecular programs (e.g., kinase/phosphatase imbalance versus broad excitatory suppression) rather than quantitative variations along a shared pathway. This necessitates sex-stratified approaches to drug development, as therapies targeting shared pathways may fail to address sex-specific mechanisms. Third, they provide mechanistic explanations for clinical observations: the concordance between female-specific oxytocin signaling suppression and heightened social stress sensitivity in women, and the association between mitochondrial dysfunction and treatment-resistant depression.

While murine models necessitate cautious extrapolation, the conservation of these pathways in human depression is supported by recent multi-omics studies implicating oligodendrocyte dysfunction and Mef2c regulation in mood disorders [54–56]. The overlap between our findings and human data strengthens the translational potential of our work, particularly regarding mitochondrial and glial mechanisms.

Limitations of this study include the focus on a single stress paradigm (SCVS), which, while validated for inducing anhedonia, may not capture all facets of chronic stress exposure. Additionally, while snRNA-seq provides transcriptional resolution, it lacks spatial context-future studies incorporating spatial transcriptomics could illuminate regional differences in sex-specific stress responses. Furthermore, causal relationships between identified molecular changes and behavioral phenotypes remain to be established through targeted manipulations. Finally, an important consideration when interpreting the implications of this study is the specific role of the hippocampus. Our findings reveal significant, sexually dimorphic molecular events in the hippocampus triggered by sub-chronic stress. However, the hippocampus is primarily implicated in the integration of emotion and cognition, whereas the core mechanisms of canonical anhedonia reside within the mesolimbic reward system (e.g., nucleus accumbens and prefrontal cortex). Consequently, our results may more accurately reflect the widespread molecular signature of the stress state or represent indirect upstream regulatory mechanisms for anhedonia. Future studies would benefit from simultaneously investigating the hippocampus and reward-related brain regions to more comprehensively map the neural circuits and molecular foundations of stress-induced anhedonia. It is important to acknowledge the limitations of our stress paradigm. While the SCVS model effectively and rapidly induces a core depressive phenotype (anhedonia) with a pronounced female bias, its relatively short duration (six days) does not fully recapitulate the chronicity of human MDD, which persists for months or years. Therefore, our findings likely capture the early, sex-specific molecular adaptations to stress that may underpin later vulnerability, rather than the sustained pathophysiology of a long-term disorder. Future studies employing longer stress durations and multiple time points are essential to trace the evolution of these molecular signatures.

Future investigations should prioritize causal validation of key mechanisms through cell-type-specific interventions, such as oligodendrocyte-targeted mitochondrial rescue or Mef2c modulation. Translational studies in human post-mortem tissue are needed to confirm the relevance of these murine findings, particularly regarding oligodendrocyte dynamics and mitochondrial gene expression. The pan-cellular upregulation of Mef2c in females positions it as a particularly promising therapeutic target warranting pharmacological exploration, potentially via epigenetic or transcriptional modulators.

Collectively, this work establishes a unified framework for understanding depression’s sex disparity and providing a roadmap for developing sex-stratified treatment strategies. By identifying cell-type-specific and sex-specific molecular targets, this study paves the way for more precise, personalized approaches to depression treatment, addressing the longstanding need for therapies tailored to the biological underpinnings of sex differences in mental health.

## Supplementary information

supplementary information

Figure S1 Quality Control Metrics Visualization for Single - Cell RNA Sequencing Data Across Different Experimental Groups.

Figure S2 UMAP plot illustrating the expression patterns of oligodendrocyte-related genes in single cells.

Figure S3 UMAP plot illustrating the expression patterns of pericyte-related genes in single cells.

Figure S4 UMAP plot illustrating the expression patterns of astrocyte-related genes in single cells.

Figure S5 UMAP plot illustrating the expression patterns of interneuron-related genes in single cells.

Figure S6 UMAP plot illustrating the expression patterns of cholinergic neurons-related genes in single cells.

Figure S7 UMAP plot illustrating the expression patterns of GABAergic neurons-related genes in single cells.

Figure S8 UMAP plot illustrating the expression patterns of glutamatergic neurons-related genes in single cells.

Figure S9 UMAP plot illustrating the expression patterns of microglia genes in single cells.

Figure S10 Venn Diagrams and Bar Plots Depicting Differential Gene Expression in Distinct Cell Types: Up - Regulated (UP) and Down - Regulated (DOWN) Genes.

Figure S11 Venn Diagrams and Supplementary Bar Plots for Up - Regulated (UP) Gene Sets Across 24 Distinct Categories (Labeled 0–23).

Figure S12 Venn Diagrams and Supplementary Bar Plots for Up - Regulated (DOWN) Gene Sets Across 23 Distinct Categories (Labeled 0–22).

Table S3 Complete Ranking of Male-Specific Up-Regulated Differentially Expressed Genes (DEGs)

Table S4 Complete Ranking of Male-Specific Down-Regulated Differentially Expressed Genes (DEGs)

Table S5 Complete Ranking of Female-Specific Up-Regulated Differentially Expressed Genes (DEGs)

Table S6 Complete Ranking of Female-Specific Down-Regulated Differentially Expressed Genes (DEGs)

Table S7 Cell number and percentage of each cell type in female and male control/experimental groups

Table S1 Key Metrics of Single - Cell Sequencing Data for Different Samples

Table S2 Gene Symbols Associated with Different Cell Types in Mouse Hippocampus and Data Sources

## Data Availability

All data reported in this paper will be shared by the lead contact upon request. All raw sequencing data generated by this study are available in the Gene Expression Omnibus (GEO) repository under the accession number GSE303470.

## Code availability

Code reported in this paper will be shared by the lead contact upon request.
