# Supplementary material for: Single-nucleus transcriptomic atlas of sexually dimorphic molecular responses to sub-chronic variable stress in the mouse hippocampus
Source: Transl Psychiatry. 2026 Jun 24;16:456. doi: 10.1038/s41398-026-04202-3 (PMC13550579; doi:10.1038/s41398-026-04202-3)
Supplement: Supplementary file 1 [file 41398_2026_4202_MOESM1_ESM.docx]

**Table S1 Key Metrics of Single - Cell Sequencing Data for Different Samples**

**Table S2 Gene Symbols Associated with Different Cell Types in Mouse Hippocampus and Data Sources**

**Table S3 Complete Ranking of Male-Specific Up-Regulated Differentially Expressed Genes (DEGs)**

**Table S4 Complete Ranking of Male-Specific Down-Regulated Differentially Expressed Genes (DEGs)**

**Table S5 Complete Ranking of Female-Specific Up-Regulated Differentially Expressed Genes (DEGs)**

**Table S6 Complete Ranking of Female-Specific Down-Regulated Differentially Expressed Genes (DEGs)**

**Table S7 Cell number and percentage of each cell type in female and male control/experimental groups**

**Figure S1 Quality Control Metrics Visualization for Single - Cell RNA Sequencing Data Across Different Experimental Groups.**

Figure S2 **UMAP plot illustrating the expression patterns of oligodendrocyte-related genes in single cells.**

Figure S3 **UMAP plot illustrating the expression patterns of pericyte-related genes in single cells.**

Figure S4 **UMAP plot illustrating the expression patterns of astrocyte-related genes in single cells.**

Figure S5 **UMAP plot illustrating the expression patterns of interneuron-related genes in single cells.**

Figure S6 **UMAP plot illustrating the expression patterns of cholinergic neurons-related genes in single cells.**

Figure S7 **UMAP plot illustrating the expression patterns of GABAergic neurons-related genes in single cells.**

Figure S8 **UMAP plot illustrating the expression patterns of glutamatergic neurons-related genes in single cells.**

Figure S9 **UMAP plot illustrating the expression patterns of microglia genes in single cells.**

Figure S10 Venn Diagrams and Bar Plots Depicting Differential Gene Expression in Distinct Cell Types: Up - Regulated (UP) and Down - Regulated (DOWN) Genes.

Figure S11 **Venn Diagrams and Supplementary Bar Plots for Up - Regulated (UP) Gene Sets Across 24 Distinct Categories (Labeled 0–23).**

Figure S12 **Venn Diagrams and Supplementary Bar Plots for Up - Regulated (DOWN) Gene Sets Across 23 Distinct Categories (Labeled 0–22).**
